# Effect of Phonotactic Constraints on Second Language Speech Processing

**DOI:** 10.1177/2041669515615714

**Published:** 2015-12-24

**Authors:** Tamami Katayama

**Affiliations:** Department of Life Science, Prefectural University of Hiroshima, Japan

**Keywords:** Second-language speech perception, phonotactics, stress

## Abstract

In this study, we examined whether phonotactic constraints of the first language affect speech processing by Japanese learners of English and whether their proficiency of the second language influences it. Native English speakers and second language speakers with a high level of language proficiency and those with a low level took part in a monitoring task. They were given two kinds of sound stimuli as target syllables (i.e., consonant–vowel and consonant–vowel–consonant) and were asked to detect them in lists of words that have stress on the first or second syllable (e.g., *biscuit* and *beside*). The results showed that both stress and phonotactics facilitated segmentation strategies by the three groups. The Japanese groups did not rely on either phonotactics or morae to segment the target syllables. They rather used stress to detect the target syllables in the English words, which is a different segmentation strategy from their first language. This study showed that phonotactic constraints did not interfere with second language processing by native Japanese speakers and provided evidence that second language speakers use the segmentation strategy that is used by native speakers of the target language.

## Introduction

### Background

Communicating with others in a second language (L2) is a necessary skill in the globalized society today. To process speech efficiently, segmenting words in the stream of speech is a vital skill as well. One of the issues in the study of speech processing is how words are segmented and how the mental dictionary, the lexicon, is accessed. Segmentation strategies for words depend on listeners' exposure to a particular language (Cooper, Cutler, & Wales, 2002). Language-specific strategies include use of suprasegmental information, such as stress and rhythm, and a segmental inventory that consists of consonants and vowels (Dupoux, Pallier, Sebastian, & Mehler, 1997). Listeners segment words on the basis of the minimal unit of their language, but it is not still clear how L2 learners segment the target language and whether they develop their own segmentation strategies. Investigation of how native and nonnative speakers auditorily detect words will contribute to the elucidation of how speech in L1 and that in L2 are processed.

Previous studies have shown that the segmentation strategies differ depending on the listener's first language. Cutler, Mehler, Norris, and Segui (1986) used a monitoring task to determine whether native English speakers (ES) perceive a phoneme or syllable itself directly. The study used seven pairs of unambiguous English content words (nouns and verbs) of similar frequencies sharing the same initial three phonemes (a consonant–vowel–consonant [CVC] sequence; e.g., *bal*), and in another context, each pair has a syllable boundary after the initial CVC (e.g., *balcony*). In the other context, the third phoneme was ambisyllabic, that is, the second consonant of the initial CVC could be considered to belong to both syllables (e.g., *balance*). Native ES were asked to give responses as quickly as possible to the target syllable when presented alongside a list of words. By measuring response time (RT), the authors examined perceptual word segmentation by syllables. The results for English-speaking subjects showed no sign of a syllabification effect, which was found in another study with French subjects (Mehler, Dommergues, & Frauenfelder, 1981). Cutler et al. concluded that segmentation strategies in continuous speech perception might well be language-specific and thus the syllable's function in speech segmentation differs depending on the speaker's languages. Mehler et al. suggested that native French speakers syllabified regardless of whether they were listening to familiar French words that were easy to syllabify or unfamiliar English words that were hard to syllabify. On the other hand, native ES did not syllabify in the same way.

If listeners segment words based on the unit of syllables, phonotactics is likely to affect speech processing. Phonotactics of every language has restrictions “on which phonemes and sequences of phones are permissible in different syllabic position” (McQueen, 1988, p.24). According to McQueen, the restriction of a phonotactic sequence differs depending on the language and it marks the starting and ending points of syllables, which is highly correlated with word boundaries. McQueen (1988) examined the effects of phonotactics and stress on speech segmentation by Dutch listeners. Forty monosyllabic Dutch words were embedded in nonsense words in the initial positions (e.g., *pil* in [pil.vrem]), which had four different conditions. One context had a syllable boundary that was aligned with the offset of the target word followed by strong stress (StrongStrong, Aligned, such as in [pil.vrem]), and another context was the same but with phonotactic constraints of the consonant sequence with a syllable boundary being misaligned with the offset of the target word (StrongStrong, Misaligned, such as in [pilm.rem]. In the third and fourth contexts, the second strong syllables were replaced with the weak vowel schwa, respectively, (StrongWeak, Aligned, as in [pil.vrəm], and Strong Weak, Misaligned, as in [pilm, rəm]). Phonotactic constraints differed under the Aligned and Misaligned contexts depending on the final phoneme of the target. In addition, another 40 words were used as targets in the final positions of nonsense target words. As well as the first targets, the second ones had four conditions depending on whether the preceding vowels are stressed or not and whether the pnototactics of the target onset is legal or not between the two vowels: StrongStrong, Aligned, as in [fim.r.ɒk]; [fim.rɒk]; StrongStrong, Misaligned, as in [fi.drɒk]; Weak Strong, Aligned, as in [fəm.rɒk]; and WeakStrong, Misaligned, as in [fə.drɒk]. The Dutch participants were instructed to spot the target words embedded in a list of nonsense bisyllables, press a button as fast as possible when they found them, and say aloud what words they heard. McQueen analyzed the RTs and the number of errors (ERR) the participants made. The Dutch speakers in his experiments failed more frequently to detect words that were misaligned with syllable boundaries cued by phonotactic constraints than to detect words that were aligned with such boundaries. Furthermore, the participants were affected by the targets in the final position, where phonotactic cues preceded the targets' onsets, more than the targets in the initial position, where the cues followed the targets' offset. McQueen suggested that phonotactic legality is taken into consideration and helps listeners to segment words. He also stated that phonotactic constraints are likely to be one of the sources of information, such as silence and metrical cues, when listeners segment words.

In addition to the effects of syllables, Cutler and Norris (1988) examined how stress affects native ES since English is a stress-based language. They tested their model of segmentation based on strong syllables. In the model of speech segmentation in a stress language, the occurrence of a strong syllable triggers segmentation of the speech signal, while that of a weak syllable does not. In their study, the participants were instructed to detect real words in nonsense strings. For example, *mint* embedded in *mintayve* and *mint* embedded in *mintesh* were presented to the listeners. Because the second syllable (*tayve*) in *mintayve* is strong, lexical access starts at *tayve*. When *mint* belongs partly to both accompanying syllables, this inappropriate intersyllabic segmentation interferes with the detection of the word *mint*. Since the weak second syllable in *mintesh* does not hinder segmentation, Cutler and Norris speculated that listeners would be able to detect *mint* faster in *mintesh* than in *mintayve*. The results supported their hypothesis that the detection of a word is delayed when the word consists of two strong syllables but not when it consists of a strong syllable followed by a weak syllable. Cutler and Norris explained that the strong syllable triggered a segmentation effect. Thus, the detection of the word was delayed in intersyllabic segmentation because the listener needed to collect speech information across a segmentation point. The results of that study supported the prediction by Mehler et al. (1981) of a stress-based segmentation model, which argues that a syllable forms a unit of speech processing.

To test the rhythmic segmentation hypothesis (Cutler & Carter, 1987; Cutler & Norris, 1988), Cutler and Butterfield (1992) used errors of spontaneous misperception. They found that strong syllables tend to be the initial syllables of lexical words, while weak syllables are more likely not to be word-initial or, if so, are more probably grammatical words. They concluded that listeners use rhythmic segmentation when perception is difficult. Filippi, Gingras, and Fitch (2014) examined prosodic effects on word learning using an Extraction or Inference or Mapping. They provided Austrian students at the University of Vienna with four conditions: consistent co-occurrence of the target label with the corresponding image category (*Co-occurrence*), *Co-occurrence* with constantly exaggerated pitch, that with randomly highlighted pitch, and that with increased duration of the target label. The participants showed a significantly higher level of performance when pitch was constantly prominent on the target. Filippi et al. suggested that pitch is more influential than duration but that attentiveness of pitch enhancement of the target label is not sufficient in learning new words. According to Filippi et al., in addition to statistical learning of the regular input, exposure to prosodically highlighted stimuli-accelerated language learning.

If listeners segment words on the basis of the rhythmic pattern in their first language, native Japanese speakers are assumed to employ the unit of mora for their segmentation strategy. According to Ladefoged (2001), “Japanese may be analyzed in terms of the classical Greek and Latin unit called mora. A mora is a unit of timing. Each mora takes about the same length of time to say” (Ladefoged, 2001, p.233). Japanese is called “mora-timed language” because its basic unit is the mora (Kubozono & Ota, 2008). Although researchers in psycholinguistic studies of speech perception have agreed on the importance of the mora as a unit of Japanese language, its role in timing remains a matter of dispute (Warner & Arai, 2001). Warner and Arai (2001) offer several hypotheses to account for timing by morae (rather than by stress); one of them is that morae are utilized to normalize word duration. Another feature of mora in Japanese is that a nasal consonant (N) is considered as one mora as well as the combination of a consonant and a vowel (CV). Otake, Hatano, Cutler, and Mehler (1993) examined the perception of segmentation of spoken Japanese words by native and nonnative listeners using a nasal in the coda position. They found, as expected, that the patterns of performance by native and nonnative listeners differed, and they confirmed the theory that speech segmentation is language-specific. Their results showed that the response patterns to CV targets were identical in CVCVCV and CVNCV (a consonant–vowel–nasal–consonant–vowel sequence) words, being inconsistent with the prediction of syllable-based segmentation. They argued that the pattern of the response by Japanese listeners can best be described in terms of mora-based segmentation because the initial mora was CV in both CVCVCV and CVNCV words. Otake et al. also claim that the mora hypothesis explains why the response pattern to CVN targets differed across CVNCV words and CVCVCV words. The Japanese listeners responded to CVN targets in CVNCV, but the RT was comparatively long. According to Otake et al., this is due to a complex target, two-mora words, while CVN targets in CVCVCV words received a mixed response. Otake et al. reported that this was predicted by the mora hypothesis because the target and stimulus do not match at the mora level. They concluded that Japanese listeners did not decompose the spoken words into syllables but into morae and that Japanese listeners thus use morae to segment speech. These results were supported by the results of Cutler and Otake's study (1994), which confirmed the theory that Japanese listeners segment speech sound targets on the basis of morae and that mora-based segmentation is language-specific, as are French listeners’ syllabic segmentation and English listeners’ stress-based segmentation. Cutler and Otake argue that Japanese listeners map their moraic pattern of speech processing onto whatever foreign languages they are learning. They therefore believe that their findings about language-specific processing have critical implications for clarifying the processes of L2 acquisition.

A bilingual study by Cutler, Mehler, Norris, and Segui (1992) provided further evidence that word segmentation strategies are language-specific. They examined how French–English bilinguals segment word strings in both English and French. Once the participants had been judged as having native-speaker competence in both languages by native speakers of French and by native speakers of English, they were asked about their language preference in order to divide them into English-dominant bilinguals and French-dominant bilinguals. Cutler et al. used the stimuli created by Mehler et al. (1981) for the French experiment and the stimuli created by Cutler et al. (1986) for the English experiment. As used in the study by Cutler et al. (1986), the bilingual participants were asked to spot words as quickly as possible when they heard the target syllables when presented alongside a list of words. As in the study by Cutler et al., the study used seven pairs of unambiguous English content words (nouns and verbs) of similar frequencies sharing the same initial two or three phonemes (CV or CVC; e.g., *ba* and *bal*). In one context, a syllable boundary was placed after the initial CVC (e.g., *balcony*). In the other context, the boundary was ambiguous because the coda (the second consonant) of the initial CVC could be considered to belong to both syllables (e.g., *balance*). The results showed that English-dominant bilinguals produced exactly the same pattern as that produced by monolingual ES not only when listening to English but also when listening to French, while the results for French-dominant participants were exactly the same as those for French speakers when the French-dominant participants listened to French materials and were exactly as those for English monolingual speakers when they listened to English. Cutler et al. argue that syllabic segmentation is a restricted procedure and can be “switched off” when it is inefficient. On the other hand, they claim that stress-based segmentation (i.e., English in this case) is an unrestricted (a general) procedure and is generally available to all speakers. Thus, while French-dominant bilinguals employed stress-based segmentation when they were presented with input in English, the reverse process is not possible, since English does not encourage the development of syllabic segmentation. Cutler et al. explain that English-dominant bilinguals use only generally available processing procedures for segmentation. They argue that the right input at the right time is required to develop a restricted processing procedure. Consequently, according to this view, even the most skilled bilingual speakers will be restricted one segmentation procedure. They therefore concluded that rhythmically based segmentation procedures are mutually exclusive as well as language-specific and that they are restricted in their availability. In this one particular aspect of language processing, even bilinguals may be functionally monolingual.

In summary, French speakers segment speech on the basis of the syllable, while Japanese speakers use the unit of mora. Although English and Dutch languages are stress-controlled languages, different segmentation strategies are adopted (Cooper et al., 2002). Rhythmic segmentation using a metrical prosody plays an important role in segmenting English speech, and lexical stress does not facilitate speech segmentation. Furthermore, the study on bilinguals shed light on how human speech processing is restricted to the dominant language. In particular, a rhythmically based procedure is incompatible with procedures for other languages, especially procedures that are syllabic in structure. Thus, speech segmentation can be considered as language-specific. Although Cutler and Otake (1994) claim that a listener's segmentation strategy in L1 is transferred into that in L2, there has not been much research on transfer of word segmentation strategies to L2. Cutler and Otake reported that native Japanese speakers segmented speech based on mora, but their methodology employed Japanese words as material. We have evidence that L2 learners develop their perception at the phoneme level. Studies on perception of L2 phonemes have revealed that the perception develops as the L2 proficiency improves (Rochet, 1995). Flege, Bohn, and Jang (1997) provided further evidence of a link between an L2 learner's perception of English vowels and the learner's L2 experience in general. They reported that adult L2 learners were able to perceive certain L2 vowels more accurately as they gained experience of their L2 language but that the L1 background did play a role in the accurate perception of L2 vowels. According to Leather and James (1991), learners adjust their perception of the target language by subsequently creating their own prototypes. However, previous studies have mainly focused on segmental level, although a unit of speech is the syllable level. Thus, it is not still clear whether perception of L2 syllable structure develops and how it affects oral communication in L2. Investigation of how words or syllables are segmented using materials in L2 is needed to unveil the development of L2 speech processing.

### Hypotheses and prediction

The aim of this study was to determine the effects of phonotactic constraints of the first language on L2 acquisition. The following research question was raised: How do native English speakers and L2 speakers of different levels in English proficiency identify target syllable units (i.e., CV and CVC) in bisyllabic or trisyllabic English words? Otake et al. (1993) reported that native Japanese speakers used the unit of mora to segment words in speech, and CVC is an illegal segmental sequence in Japanese except for the case in which the coda is nasal. Therefore, I predicted that the Japanese groups would segment CV syllables faster than CVC syllables. In addition, since Cutler and Norris (1988) reported that native ES used stressed syllables to segement words, the factor of stress was also included in this study and the effects of phonotactics were compared. Japanese accents are realized by pitch rather than stress. Native Japanese speakers recognize a word accent on the syllable on which pitch falls (Kubozono, 1998). Although stress facilitates segmentation of continuous speech by native English speakers, this is not the case with native Japanese speakers. Thus, the following hypotheses were raised:

1. Since Japanese is not a stress-timed language, placement of stress makes no difference to Japanese learners of English (and less so for Japanese speakers with low proficiency of English than for those with high proficiency) for syllable recognition in English.
2. Since most of Japanese moras consist of CV units, Japanese learners of English will recognize CV units faster than CVC units.

Based on these hypotheses, native English speakers and L2 speakers with a high level of language proficiency and those with a low level took part in a monitoring task.

## Experiment: Monitoring Task

### Materials

Twenty bisyllabic or trisyllabic words with target syllables (CVC or CV) were selected as target words (see Table 1). Half of them have stress on the first syllables, and the others have stress on the second syllables. Thus, stimuli under the following four conditions were created.

1. Target words have stress on the first syllables whose structure is the same as the target syllable structure: a target word is *biscuit* and its target syllable is *bis* (CVC).
2. Target words have stress on the first syllables whose structure is partly the same as the target syllable structure; a target word is *biscuit* and its target syllable is *bi* (CV).
3. Target words have stress on the second syllables and the target syllables are placed over the first syllables and the second syllables in a word; a target word is *beside* and its target syllable is *bis* (CVC).
4. Target words have stress on the second syllables whose structure is the same as the target syllable structure; a target word is *biscuit* and its target syllable is *bi* (CV).

**Table 1.** Target Words and Syllables.

No.	Stressed syllable	Target word	Version I	Version II	Stressed syllable	Target word	Version I	Version II
1	first	biscuit	bis	bi	second	beside	bi	bis
2		bister	bi	bis		besiege	bis	bi
3		Bigfoot	big	bi		begin	bi	big
4		bicker	bi	bik		became	bik	bi
5		piddle	pid	pi		pedometer	pi	pid
6		picnic	pi	pik		pecan	pik	pi
7		mote	mote	mou		motel	mou	mote
8		niggle	ni	nig		neglect	nig	ni
9		picture	pik	pi		peculiar	pi	pik
10		mosaic	mouz	mou		mosey	mou	mouz

In addition to the target words, 20 distractors (the first consonants being the same as the target syllables but the following vowels being different) and 400 fillers were selected, and the ratios of words with stress on the first syllables and words with stress on the second syllables were 50%. Two versions of a monitoring task were created with the aid of E-prime 2.0 software (2012) for ES and Japanese speakers. The instructions were in English for the ES and in Japanese for the Japanese speakers. Each version of instructions had 40 lists including 20 positive lists with the target words and 20 negative lists without them. Each list had eight to twelve words. Each positive list had one target word and the rest of the spaces were occupied by fillers, and each negative list consisted of a foil and fillers. The target words had stress either on the first or second syllables, and they were each presented ten times. All of the conditions for the English and Japanese versions were the same except for the target syllables. The different types of segmental sequences (i.e., CV and CVC) were presented as target syllables in the two versions. For example, when a target word was *biscuit, bis* was presented in version I and *bi* was presented in version II. The proportions of different types of target words (i.e., different placement of stress) and target syllables (i.e., CV and CVC) were counterbalanced in each list.

A target syllable was set up to be presented auditorily followed by a blank screen for 750 ms. Then the words in the list were programmed to run while the mark “+” appeared in the center of the screen. If a response was made while the words in a particular list were being presented, this list was programmed to jump to the next list. If there was no response by the end of the list, the screen informed the participant of the end of list and instructed the participant to press the button to continue.

### Participants

Twenty native English speakers, 19 Japanese speakers with high proficiency of English (JH), and 20 Japanese speakers with relatively low English proficiency (JL) took part in the experiment. ES were native English speakers in the Boston area with a mean age of 48.5 years. Most of the JH were English or Japanese instructors at a college or at a private English conversation school, and their mean scores for TOEIC and TOEFL (PBT) were 862 and 600, respectively. Their mean age was 33.8 years and the mean period of living in English-speaking countries was 2 years and 7 months. JL were English learners at the university level in Japan, and their ages ranged from 19 to 22 years. None of them had lived overseas. All of the participants reported that they had no hearing impairment.

### Procedure

The participants were instructed to sit in front of the computer in a quiet room and put on the headphones. Instructions were presented on the screen, and they were asked to respond as quickly as possible by pressing button “1” on the response box when they heard the target syllable in a word. The RT from the onset of each target word was recorded. When they did not hear the target syllable, they were instructed to press button “5” to continue. The target syllable and lists of words were presented after the auditory instruction “Please listen for.” Each word in the list was followed by a 1,000-ms interval, and the next words were presented automatically.

## Results

**Figure 1.**
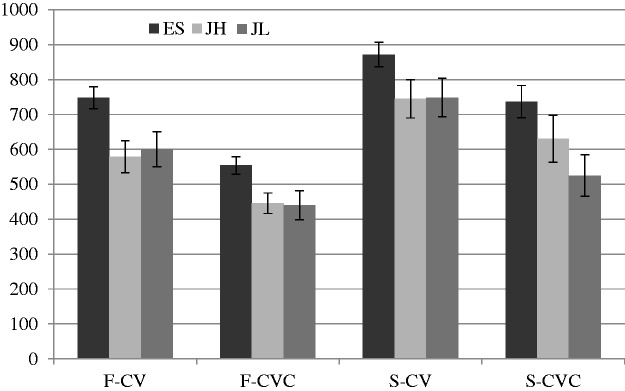
Mean responses times in the three groups are shown. F indicates target words with stress on the first syllables and S indicates target words with stress on the second syllables. ES: English speakers, JH: Japanese speakers with high proficiency of English, JL: Japanese speakers with low proficiency of English; CVC: consonant–vowel–consonant; CV: consonant–vowel.

**Figure 2.**
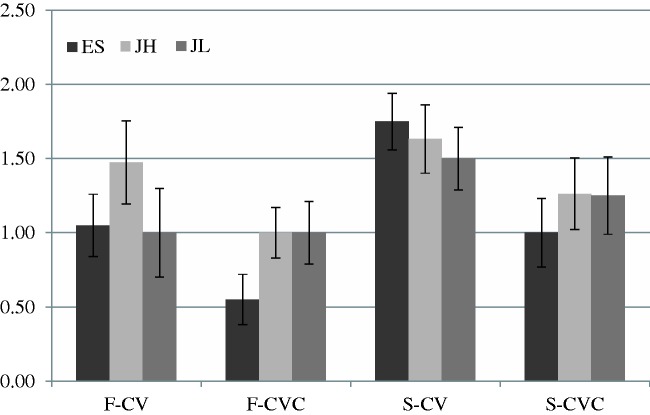
Mean numbers of errors in the three groups are shown. F indicates target words with stress on the first syllables and S indicates target words with stress on the second syllables. ES: English speakers, JH: Japanese speakers with high proficiency of English, JL: Japanese speakers with low proficiency of English; CVC: consonant–vowel–consonant; CV: consonant–vowel.

Each participant listened to the target words under four conditions. The duration of target syllables was subtracted from the response time (RT) for analysis. Figure 1 shows RT for the four conditions in the three groups. The mean numbers of errors (ERR) was also counted. Figure 2 shows ERR when the participants were given four target words. Two-way repeated measures ANOVA was performed for RT and ERR in the three groups. The esults for ES indicated significant effects of stress and phonotactics both in RT, stress: *F*(1, 19) = 29.47, *p* < .001, η^2^ = .61; phonotactics: *F*(1, 19) = 36.35, *p* < .001, η^2^ = .66, and ERR, stress: *F*(1, 19) = 6.74, *p* < .05, η^2^ = .26; phonotactics: *F*(1, 19) = 11.05, *p* < .01, η^2^ = .37. The results for JH showed significant effects of stress and phonotactics in RT, stress: *F*(1, 18) = 29.59, *p* < .001, η^2^ = .62; phonotactics: *F*(1, 18) = 24.00, *p* < .001, η^2 = ^ .57, but there were no effects in ERR, stress: *F*(1, 18) = 1.15, *p* = .30, η^2^ = .60; phonotactics: *F*(1, 18) = 2.57, *p* = .13, η^2^ = .13. The results for JL also indicated significant effects of stress and phonotactics in RT, stress: *F*(1, 19) = 13.58, *p* < .01, η^2^ = .42; phonotactics: *F*(1, 19) = 52.38, *p* < .001, η^2 = ^ .73, but only the effect of stress was significant in ERR, stress: *F*(1, 19) = 4.67, *p* < .05, η^2^ = .20; phonotactics: *F*(1, 19) = .27, *p* = .061, η^2^ = .01. There were no significant interactions between stress and phonotactics in the three groups in RT, ES: *F*(1, 19) = 1.03, *p* = .32, η^2^ = .05; JH: *F*(1, 18) = .15, *p* = .70, η^2^ = .008; JL: *F*(1, 19) = 1.48, *p* = .24, η^2^ = .07, or in ERR, ES: *F*(1, 19) = .35, *p* = .56, η^2^ = .02; JH: *F*(1, 18) = .10, *p* = .76, η^2^ = .008; JL: *F*(1, 19) = .22, *p* = .64, η^2^ = .01.

The results revealed that the participants detected the target words significantly faster and more accurately when they were given the target syllable CVC (mean response time [*M* RT] = 555.4 ms, mean ERR [*M* errors] = 1.0) than when they were given the target syllable CV (*M* RT = 715.2 ms, *M* errors = 1.4). In addition, RT was significantly faster and ERR was smaller when stress was on the first syllable (*M* RT = 561.2 ms, *M* errors = 1.0) than when it was on the second syllable (*M* RT = 709.5 ms, *M* errors = 1.4). JL responded to the words significantly faster (*M* = 578.4 ms) than did ES (*M* = 727.6 ms), but the difference between RT of JL and JH (*M* = 600.1 ms) was not significant. Target words were missed most frequently by JH (*M* = 1.3), second most frequently by JL (*M* = 1.2), and least by ES (*M* = 1.1), though the differences were not significant.

In short, all of the groups tended to find the target words faster and more accurately when the given target syllable structure was CVC and the target words to be identified had stress on the first syllables. Moreover, ES responded to the target words slowest but most accurately among the three groups. Compared with the results for JH, JL tended to identify the targets faster and more accurately, though the differences were not statistically significant.

## Discussion

This study was carried out to determine the effects of phonotactic constraints of the first language on acquisition of a L2 by examining how native English speakers and L2 speakers of different levels in proficiency segment target syllable units (i.e., CV and CVC) in bisyllabic or trisyllabic English words with stress either on the first or second syllables. The following two hypotheses were raised:

1. Since Japanese is not a stress-timed language, placement of stress makes no difference to Japanese learners of English (and less so for Japanese speakers with low proficiency of English than for those with high proficiency) for syllable recognition in English.
2. Since most of the Japanese morae consist of CV units, Japanese learners of English will recognize CV units faster than CVC units.

It was found that participants in all of the groups responded to the target words faster when stress was on the first syllables and the target syllable structure was CVC. ES identified the target syllables the most accurately but the slowest. JL showed better performance both in accuracy and speed than JH. The Japanese groups did not rely on either phonotactics or mora to segment the target syllables. They rather used stress to detect the target syllables in the English words, which is a different segmentation strategy from that of their L1. Therefore, both hypotheses were rejected in this study. However, there is a limitation in the methodology of this study. Some given target syllables were ambiguous as to whether they were [big] or [bik] because the stop consonants were not pronounced clearly in the coda position. In addition, the difference in sound quality between stressed syllables and unstressed syllables was likely to have affected the results. Since the target syllables were recorded separately, they were pronounced with stress. The target syllables were embedded in either stressed syllables or the unstressed syllables in the target words, and thus the sound quality was not exactly the same. Further study is needed to control these variables.

The results for ES were consistent with those in a study by Mehler et al. (1981) with respect to the fact that stress and phonotactics facilitate ES' segmentation strategies. ES found it difficult to detect the target syllables in the words when they were unstressed despite the matched target syllables (e.g., [bi] in ***be**siege*). These results provide further evidence that stress facilitates native ES' segmentation. In addition, ES found it easier to identify the target syllables (e.g., [bis]) when the coda of the target syllable (e.g., [s] of [bis]) crossed the second syllable with stress (e.g., ***bes**iege*) than when the target syllable (e.g., [bi]) was a part of the stressed first syllable (e.g., ***bi**ster*). A syllable is the minimal unit of speech processing and English does not have a monosyllabic word pronounced [bi]. That is, CV units (e.g., [bi]) are ill-formed syllables unlike Japanese, while CVC units are well formed in English. Thus, it was likely that ES did not match the target syllables (CV) in words. Although their accuracy was the best among the three groups, their RT was the longest among the groups. Since English words were used in this study, ES might have tried to match the target syllable and the corresponding words while considering possible acoustic variations. According to Jusczyk (1997), innately guided learning enables infants to pay attention to particular acoustic information more than others in order to process their L1 speech effectively. Although they can perceive various types of acoustic information that surrounds them, they choose only necessary information to create the categories of their L1 phonemes. Thus, ES were likely to have put the allophones into the same category while selecting the critical information. In this sense, word familiarity was likely to have affected the results since some words (e.g., *picture* or *became*) are common, while others (e.g., *bister* or *piddle*) are not common. A possible reason why ES responded the most slowly is aging effect. The Japanese groups, especially JH, were younger than ES. Although the aging effect on RT has not been proved in previous studies, further study with control of these variables is needed.

On the other hand, JL responded to the words fastest, but they missed the most among the three groups. Since they do not have sufficient linguistic knowledge to access a mental lexicon in L2, it is assumed that they focused on only acoustic information to agree with that of the target syllables in the words and thus reacted quickly. At the same time, however, it is assumed that their lack of sufficient L2 phonological categories to cover acoustic variations was responsible for their failure to find target syllables that have only slight acoustic differences. Compared with JL, JH have advanced L2 knowledge and have developed phonological categories to deal with English sound variations. Thus, more time is required to connect the acoustic input and their linguistic knowledge, which might have caused JH to respond to the targets more slowly than JL.

JL and JH showed the same tendency as that of ES with respect to RT and ERR. The results showed that JL and JH responded faster and more correctly when they segmented CVC syllables than when they segmented CV syllables. This indicates that they neither used a segmentation strategy based on mora-timing or the Japanese syllable structure. Since Otake et al. (1993) reported that native Japanese speakers used morae to segment words in speech, I predicted that the Japanese groups would segment CV syllables faster than CVC syllables. However, they responded to CVC syllables faster regardless of the placement of stress (even when the coda of the target syllable crossed the second syllable (e.g., [bis] in ***bes**iege*)). The reason why JH and JL did not rapidly respond to CV syllables might be that acoustic information was not sufficient to detect the target words. The stimuli [bi] and [pi] could be acoustically ambiguous without context, but [bis] has additional information of the coda [s]. This allows the participants to specify the target syllables and to find the target words easily. When English words were used as stimuli in this study to investigate L2 speech processing, the results were not consistent with those in a study by Otake et al. (1993). In addition, both JL and JH responded more rapidly and more accurately to words with stress on the first syllables than to words with stress on the second syllables, indicating that stress facilitated detection of target syllables. They used stress to segment the target syllables as the native ES did. It is assumed that stress makes the syllables acoustically prominent, helping the Japanese speakers to find the target easily. Again, target syllables were pronounced separately for the recording and thus they were produced as stressed syllables. This is likely to have allowed the Japanese groups to match the words with stress on the initial syllables better than words with stress on the second syllables. Another possible reason is that stress on the first syllable is more common in English, so that the Japanese groups found it easy to segment syllables in the words. Cutler et al. (1986) claim that rhythmically based segmentation procedures are mutually exclusive as well as language-specific and that they are restricted in their availability. The results of this study, however, showed that the Japanese speakers did not use their L1 segmentation strategy to segment target syllables in English words regardless of their L2 proficiency. Although they use morae for speech processing in L1, this strategy can be adjusted when they process L2 speech, and thus phonotactic constraints did not hamper the Japanese groups' segmentation strategies in this study.

Perception of L2 phonemes develops as learners gain more experience, but this was not the case with L2 phonotactics. There was not a significant difference depending on L2 proficiency when they segmented the syllables of the target language. The results suggest that the native Japanese speakers employed not their moraic pattern but English stress pattern for L2 speech processing, which was different from the prediction. In short, phonotactics of the L2 was perceived by the learners regardless of their L2 experience unlike phonemes, and phonotactic constraints of L1 do not prevent them from segmenting L2 speech. These results provided additional evidence to reveal how learners process a second language and what aspect they develop for speech processing. However, the findings of this study raised some fundamental questions as to whether speech processing strategies are language-specific or not. If they are, further study is required to examine how native Japanese speakers segment words both in L1 and L2.

## Conclusion

In conclusion, this study showed that phonotactic constraints did not interfere with L2 processing by native Japanese speakers and provided evidence that L2 speakers use the segmentation strategy that is used by native speakers of the target language. Further investigation is needed to determine how native Japanese speakers segment a syllable in a nonword and to determine whether there is a difference between groups with different levels of English proficiency. Results of such studies may help to reveal the mechanism of L2 speech processing.
